# Simultaneous mapping of membrane voltage and calcium in zebrafish heart *in vivo* reveals chamber-specific developmental transitions in ionic currents

**DOI:** 10.3389/fphys.2014.00344

**Published:** 2014-09-11

**Authors:** Jennifer H. Hou, Joel M. Kralj, Adam D. Douglass, Florian Engert, Adam E. Cohen

**Affiliations:** ^1^Department of Physics, Harvard UniversityCambridge, MA, USA; ^2^Department of Chemistry and Chemical Biology, Harvard UniversityCambridge, MA, USA; ^3^Department of Molecular and Cellular Biology, Harvard UniversityCambridge, MA, USA; ^4^Howard Hughes Medical InstituteChevy Chase, MD, USA

**Keywords:** voltage imaging, cardiac development

## Abstract

The cardiac action potential (AP) and the consequent cytosolic Ca^2+^ transient are key indicators of cardiac function. Natural developmental processes, as well as many drugs and pathologies change the waveform, propagation, or variability (between cells or over time) of these parameters. Here we apply a genetically encoded dual-function calcium and voltage reporter (CaViar) to study the development of the zebrafish heart *in vivo* between 1.5 and 4 days post fertilization (dpf). We developed a high-sensitivity spinning disk confocal microscope and associated software for simultaneous three-dimensional optical mapping of voltage and calcium. We produced a transgenic zebrafish line expressing CaViar under control of the heart-specific *cmlc2* promoter, and applied ion channel blockers at a series of developmental stages to map the maturation of the action potential *in vivo*. Early in development, the AP initiated via a calcium current through L-type calcium channels. Between 90 and 102 h post fertilization (hpf), the ventricular AP switched to a sodium-driven upswing, while the atrial AP remained calcium driven. In the adult zebrafish heart, a sodium current drives the AP in both the atrium and ventricle. Simultaneous voltage and calcium imaging with genetically encoded reporters provides a new approach for monitoring cardiac development, and the effects of drugs on cardiac function.

## Introduction

The cardiac action potential (AP) arises through the interaction of a large number of membrane proteins, and thus is an essential indicator of cardiac function. The action potential depolarization causes voltage-gated Ca^2+^ channels to open. The inward flow of Ca^2+^ then initiates calcium-induced calcium release from the sarcoplasmic reticulum. The rapid spike in cytosolic Ca^2+^ causes muscular contraction. Improved understanding of cardiac physiology and development (Panáková et al., 2010) requires new approaches to measure the relation of electrical and calcium dynamics, with wide dynamic range in space and time, and capability for long-term measurements *in vivo* (Kaestner and Lipp, 2011; Mandel et al., 2012).

Voltage-sensitive dyes (VSDs) have been used to study AP waveforms from excised animal hearts since the 1970's (Salama and Morad, 1976; Entcheva and Bien, 2006; Panáková et al., 2010). However, due to dye-mediated phototoxicity, optical recordings with VSDs typically do not extend beyond 1 min, and preparations are not stable for repeated imaging. Dye-mediated phototoxicity is most acute for high-magnification single-cell imaging, due to the high illumination intensity needed to produce sufficient fluorescence signal from a small field of view. The difficulty of targeting dyes to specific cell types presents a challenge for cellular-resolution voltage imaging *in vivo*.

Zebrafish are a promising model for cardiac development on account of their small size, genetic tractability, and transparency (Milan et al., 2003, 2009). For measurements *in vivo*, genetically encoded reporters are preferred due to reduced toxicity and the ability to target specific cell types (Tsutsui et al., 2008). Voltage imaging with a genetically encoded voltage indicator (GEVI) in zebrafish hearts was first performed with a FRET-based sensor, called Mermaid (Tsutsui et al., 2010), which reported action potentials with a 2.3% change in ratio of donor to acceptor fluorescence. The step response of Mermaid shows complex temperature-and voltage-dependent multi-exponential kinetics with time constants ranging from ~10 to 600 ms. Because of its small voltage response and low speed, Mermaid imaging was not able to resolve details of the AP waveform. The blue/green spectra of the Mermaid FRET pair also precluded imaging in combination with any genetically encoded Ca^2+^ sensor.

Calcium imaging in zebrafish hearts has been performed using Ca^2+^-sensitive dyes (Sehnert et al., 2002; Milan et al., 2006) and genetically encoded indicators based on the GCaMP scaffold (Chi et al., 2008). However, spectral overlap of GCaMP reporters with previously used voltage reporters has prevented simultaneous optical measurement of voltage and calcium *in vivo*. Furthermore, previously used genetically encoded voltage indicators lacked the temporal resolution to differentiate chamber-specific action potential waveforms. Simultaneous optical recording of voltage and Ca^2+^ waveforms can provide insights into cardiac development not attainable from either modality alone.

The far-red spectrum of rhodopsin-based voltage indicators (Kralj et al., 2011, 2012) enables combined application with GFP-based reporters of calcium and, in principle, other analytes. Archaerhodopsin 3 (Arch) (Chow et al., 2010), from the Dead Sea microorganism *Halorubrum sodomense*, has a rapid voltage response (~0.5 ms), but generates a small hyperpolarizing photocurrent during imaging. The mutant Arch(D95N) does not perturb membrane potential and shows mixed kinetics at room temperature with ~20% response in ~1 ms and the remaining response in ~40 ms. Newer Arch-based voltage indicators, called QuasArs, show improved speed, sensitivity, and brightness, with no photocurrent (Hochbaum et al., 2014) but these have not yet been put into transgenic zebrafish. Arch-based voltage indicators are 50–100-fold dimmer than GFP-based variants, so these reporters require specialized optical instrumentation to achieve high-speed and high-sensitivity imaging.

Here we introduce a dual-function Ca^2+^ and voltage reporter, CaViar, based upon a fusion of GCaMP5G to Arch(D95N). In combination with custom imaging hardware and software, we used CaViar to simultaneously map AP propagation and Ca^2+^ dynamics in the embryonic zebrafish heart. We identified a chamber-specific transition during development from a Ca^2+^-dominated AP upstroke to a Na^+^-dominated AP upstroke. This transition occurred first in the ventricle, and later in the atrium. Distinct effects of drugs in the atrium and ventricle of the zebrafish heart highlight the importance of measurements in well-defined cellular subtypes *in vivo*.

## Materials and methods

### Molecular cloning

For expression of CaViar in HEK293 cells, we constructed plasmid pJMK019 comprising Arch(D95N)-GCaMP5G in a pLenti-CMV-Puro backbone. The pLenti-CMV-Puro backbone (Addgene 17448) was cut with *Bam*HI and *Sal*I restriction enzymes and isolated by gel purification. Arch(D95N) was amplified from pJMK004 (Addgene 34616) using:

Forward 5′-CCATAGAAGACACCGACTCTAGAGATGGACCCCATCGCTCTG-3′ and Reverse 5′-TGTCGGCCTTGATATAGACGTTACCGGTCGGTCGGC-3′.

GCaMP5G was amplified from pCMV-GCaMP5G (AddGene 31788) using:

Forward 5′-GCCGACCGACCGGTAACGTCTATATCAAGGCCGACA-3′ and Reverse 5′-AATTTTGTAATCCAGAGGTTGATTGTTACTTCGCTGTCATCATTTG-3′.

Arch(D95N) and GCaMP5G were joined by a 15 amino acid linker, consisting of TGSGASGSHHHHHHG. Shorter linkers appeared to disrupt the function of the GCaMP, but with this linker, the two proteins behaved as in isolation. The backbone and two genes were joined via isothermal ligation (Gibson et al., 2009) for 1 h at 50°C. Proper insertion was checked by sequencing.

For expression in zebrafish, Arch(D95N)-GCaMP5G was amplified by PCR from pJMK019 and cloned into the *Spe*1 site of a Gateway (Life Technology) destination vector, which contained Tol2 transposase recognition sequences. The *cmlc*2 promoter sequence was amplified and BP-cloned into a compatible entry vector. The resulting entry and destination clones were then LR-reacted to make *cmlc*2:Arch(D95N)-GCaMP5G expression vectors.

### High-sensitivity spinning disk confocal imaging

Confocal images were acquired on a modified Yokogawa CSU-X1 spinning disk unit attached to an Olympus IX71 inverted base. We found that the as-delivered spinning disk unit lacked sufficient sensitivity to image dim Arch(D95N) fluorescence in zebrafish heart. We made two critical modifications to maximize illumination intensity at the sample and to minimize background autofluorescence.

Traditionally, illumination is coupled into the CSU-X1 via a single-mode fiber, whose output is expanded to overfill an internal field aperture to produce uniform illumination across the field of view. Between coupling losses from the single-mode fiber and from the overfilling the internal field aperture, excitation path optical throughput was 1–2%. Furthermore, uniform illumination of the whole field of view caused the illumination intensity at the zebrafish heart (which only filled ~20% of the field of view) to be too low, given the available laser power. We sought a means to increase the illumination intensity on the relevant region of the sample.

We removed the last turning mirror before the microlens array of the CSU-X1 and directly coupled the excitation beams (wavelengths 488 and 635 nm) onto the microlens array. With this modification, 10–20% of the laser power reached the sample, a 10-fold increase over the optical fiber-based coupling method. This modification allowed user control over the size and profile of the illumination. By focusing the beam on a sub-region of the field of view a user could achieve significantly enhanced illumination intensity, at the cost of uniformity of illumination. Non-uniform illumination is a minor concern for samples that only occupy a fraction of the field of view; and furthermore is readily corrected computationally by performing a flat-field correction with an image acquired on a uniform dye sample.

**Figure 1:**
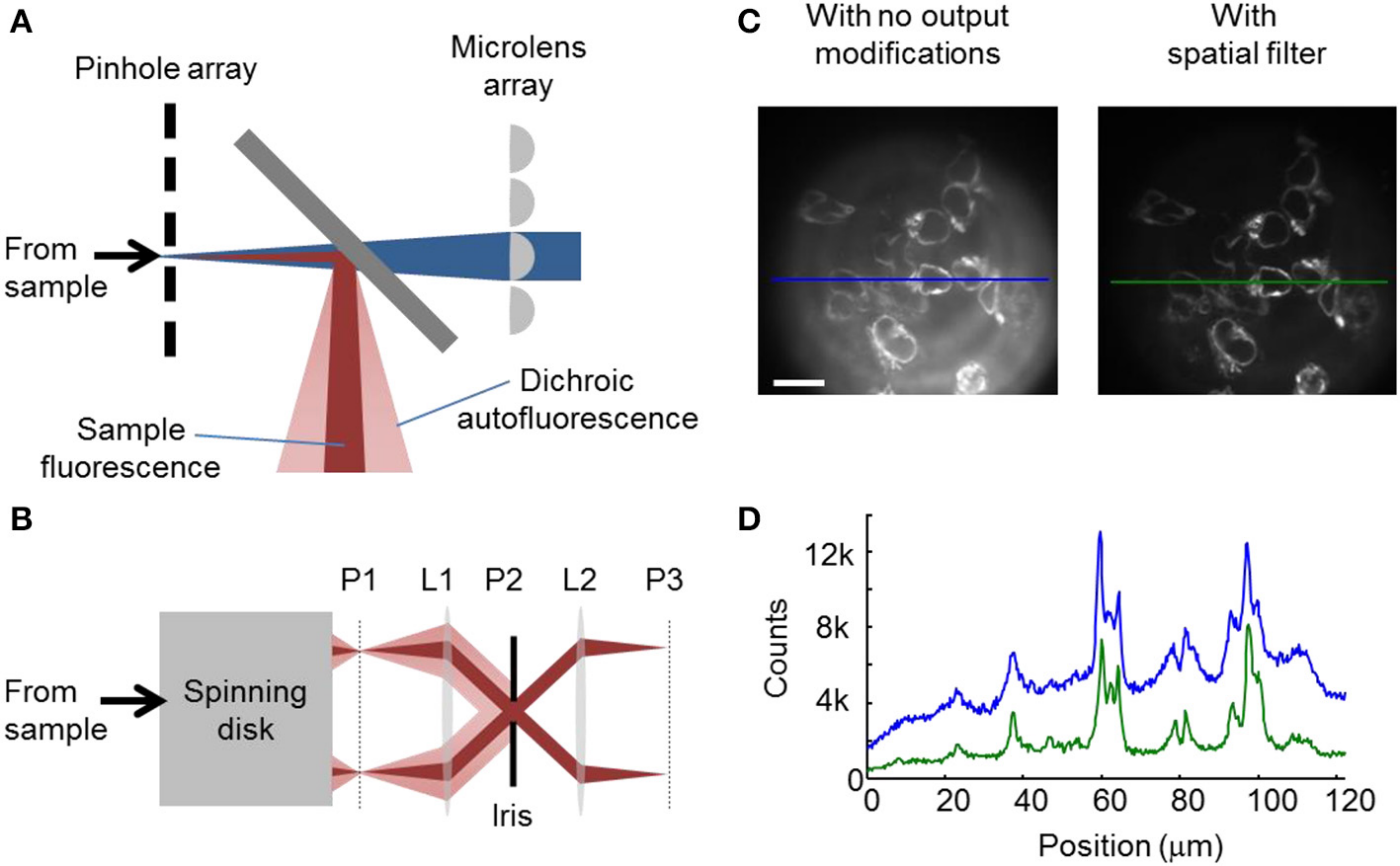
**Modified spinning disk confocal microscope for high-sensitivity imaging**. **(A)** Internal optics of the spinning disk unit. The pinhole array and the microlens array are mounted co-axially on a shaft that rotates both at a high speed (typically 10,000 rpm). The excitation light (*blue*) passes through the short-pass dichroic, from which it elicits undesirable autofluorescence *(pink*). Fluorescence returning from the sample (*red*) emerges with a narrower cone-angle than autofluorescence of the dichroic. **(B)** Spatial filter for blocking internally generated autofluorescence. The aperture at plane P2 is configured to block most autofluorescence while passing most sample fluorescence. **(C)** Image of HEK cells expressing Arch(D95N) (xλ_exc_ = 594 nm, λ_em_ > 655 nm) as imaged without the spatial filter (*left*), or with the spatial filter (*right*). Scale bar 20 μm. **(D)** Line-profiles through the indicated sections of images in **(C)**, showing the higher background without the spatial filter (*blue*) than with the spatial filter (*green*).

In the presence of higher illumination intensity than is encountered under standard operating conditions, we observed a background autofluorescence signal that overwhelmed the dim sample fluorescence. Inserting a beam block between the CSU-X1 spinning disk unit and the microscope body did not reduce this autofluorescence, implying that the autofluorescence originated within the spinning disk unit. The spatial pattern of the autofluorescence indicated that it originated from an optical element oriented at 45° relative to the optical axis. The internal CSU-X1 dichroic mirror was the only optical element with this orientation (Figure 1A). This autofluorescence interfered with imaging of very dim samples such as Arch(D95N). All dichroic mirrors tested produced some autofluorescence. This problem is more severe in spinning disk confocal microscopy than in standard epifluorescence microscopy because in spinning disk the high intensity excitation beam interacts with the bulk of the short-pass dichroic substrate, while in conventional epifluorescence the high intensity excitation beam reflects off the front surface of the long-pass dichroic and only the dim fluorescence of the sample passes through the bulk.

We took advantage of the differing angular distributions of the dichroic autofluorescence and the sample fluorescence to block most of the former while passing the latter (Figure 1B). Traditionally, the camera resides at P1, an image plane just outside the spinning disk unit. The numerical aperture of the sample fluorescence at P1 is given by:

$$NA_{samp}=NA_{obj}/M,$$

where *NA*_*obj*_ is the numerical aperture of the objective and *M* is the magnification. The numerical aperture of the autofluorescence at P1 is:

$$NA_{auto}=D_{0}/2f_{0},$$

where *D*_0_ is the diameter and *f*_0_ is the focal length of the last lens inside the spinning disk unit, assuming that this lens provides the limiting aperture on dichroic autofluorescence.

We placed a lens L1 with focal length *f*_1_, focused on P1. This lens collimated the rays emerging from each pinhole in the spinning disk into a bundle of diameter:

$$D_{1}=2f_{1}NA_{obj}/M.$$

At plane P2, a distance *f*_1_ away from L1, these bundles of rays crossed. An aperture of diameter *D*_1_ placed in plane P2 passed the light from the pinholes. The autofluorescence of the dichroic was not focused at plane P2 and thus was largely blocked by the aperture. A lens L2 focused on P2 formed an image at P3, where a camera was placed.

### Imaging conditions

Samples were illuminated by solid state lasers at 488 nm (Coherent Obis, 50 mW) with intensity 0.45 W/cm^2^ and at 635 nm (Dragon laser, 300 mW) with intensity 40 W/cm^2^. A custom dichroic (Chroma Technology, optimized for 488/640 illumination) within the CSU-X1 unit passed the excitation beams and reflected the sample fluorescence. Imaging was performed with a 20x water-immersion objective with coverslip correction (Zeiss Plan-Apochromat NA 1.0). Emission fluorescence passed through a quad-band emission filter (Chroma NC264505-ZET405/488/532/642m) and then passed through the spatial filter apparatus described above. A home-built dual-view imaging system projected emission of wavelengths <540 nm and >640 nm onto adjacent halves of an EMCCD camera (Andor iXon X3, 512 × 512 pixels). Exposure times ranged from 8 to 50 ms. A piezoelectric objective scanner (PIFOC PD72Z4CA0) set the *z*-position of the focus. A custom LabView (National Instruments) script controlled illumination, the objective scanner, and the camera for automated data acquisition.

### HEK293 cell culture

HEK293T cells were cultured and transfected as previously described in Maclaurin et al. (2013).

### Electrophysiology

Filamented glass micropipettes (WPI) were pulled to a tip resistance of 3–10] MΩ, fire polished, and filled with internal solution (containing, in mM: 125 potassium gluconate, 8 NaCl, 0.6 MgCl_2_, 0.1 CaCl_2_, 1 EGTA, 10 HEPES, 4 Mg-ATP, 0.4 Na-GTP; pH 7.3; adjusted to 295 mOsm with sucrose). The micropipettes were positioned with a Burleigh PCS 5000 micromanipulator. Whole-cell voltage clamp recordings were acquired using an AxoPatch 200B amplifier (Molecular Devices), filtered at 2 kHz with the internal Bessel filter, and digitized with a National Instruments PCIE-6323 acquisition board at 10 kHz. Ambient 60 Hz noise was removed by digital filtering during post processing.

### Zebrafish breeding

All experiments were conducted in accordance with Harvard IACUC protocols. Adult zebrafish (strain AB) were raised and bred at 28.5°C according to standard methods. Single-cell embryos were injected with a mixture of *cmlc*2:Arch(D95N)-GCaMP5G and Tol2 mRNA, each at a concentration of 30 ng/μL, and raised to adulthood. Potential founders were crossed with wild-type fish. Founders were identified by screening progeny for cardiac GCaMP5G fluorescence. F1 embryos were used for all imaging experiments.

### Imaging of zebrafish hearts

Embryos were reared in E3 medium (5 mM NaCl, 0.17 mM KCl, 0.33 mM CaCl_2_, 0.33 mM MgSO_4_) containing 0.003% 1-phenyl-2-thiourea to prevent melanization. Embryos were manually dechorionated and mounted in 2% low melting point agar dorsal-side down (40 hpf or before) or ventral-side down (after 40 hpf) on #1 glass coverslips. Heart contraction was arrested by incubating the mounted embryos in 50 μM blebbistatin for 2 h prior to imaging.

A 4 mM stock of all-*trans* retinaldehyde was made in 33% propylene glycol and 45% (2-hydroxypropyl)-B-cyclodextrin solution. For imaging fish before 48 hpf, larvae were soaked in a 1:1000 dilution of this stock in E3 for 9–12 h prior to imaging. After 48 hpf, fish produced sufficient endogenous retinaldehyde to saturate the binding pocket in Arch(D95N), and so no exogenous retinaldehyde was added.

For the pharmacological experiments, 1000x stocks of nifedipine (10 mM) and quinidine (10 and 200 mM) were made in DMSO. The stocks were mixed at a 1:1000 dilution into E3 buffer containing 50 μM blebbistatin to inhibit contraction. The agarose-mounted embryos were soaked in the resulting drug-E3 solution or vehicle control solution (0.1% DMSO, 50 μM blebbistatin in E3) for 5 h at 28.5°C prior to imaging.

### Acquisition of three-dimensional data sets

For the three-dimensional reconstruction of the zebrafish heart, pairs of consecutive *z*-focal planes, separated by 5 μm, were recorded within a single movie, with typically ~10 beats recorded in each *z*-plane. Timing differences between successive focal planes were calculated from the phase shift of the action potential waveform between the first and second halves of each movie. Blue illumination was found to inactivate blebbistatin, so for movies of GCaMP5G fluorescence, fish were kept in the dark for 7 min. between movies to allow blebbistatin to reperfuse the heart. Maps of action potential propagation within each *z*-plane were constructed via temporal registration and averaging of recordings of 10 beats. Data from all *z*-planes was combined to create a 4-D (*x*, *y*, *z*, *t*) movie of AP propagation. The boundary of each *z*-plane was segmented and used in the Matlab software package iso2mesh (Fang and Boas, 2009) for volumetric rendering of the zebrafish heart. A modified version of this software enabled color to reflect the voltage values derived from the fluorescence data.

## Results and discussion

### High sensitivity imaging with a modified spinning disk confocal microscope

Spinning disk confocal fluorescence images of Arch(D95N) fluorescence in human embryonic kidney (HEK) cells were contaminated by significant background autofluorescence produced by the dichroic mirror (Figure 1C). Insertion of the spatial filter apparatus of Figure 1B into the imaging path decreased background autofluorescence 3.7-fold, with only 1.25-fold decrease in sample fluorescence (Figures 1C,D). This setup was essential for ultrasensitive three-dimensional fluorescence imaging.

### Characterization of CaViar in HEK cells

**Figure 2:**
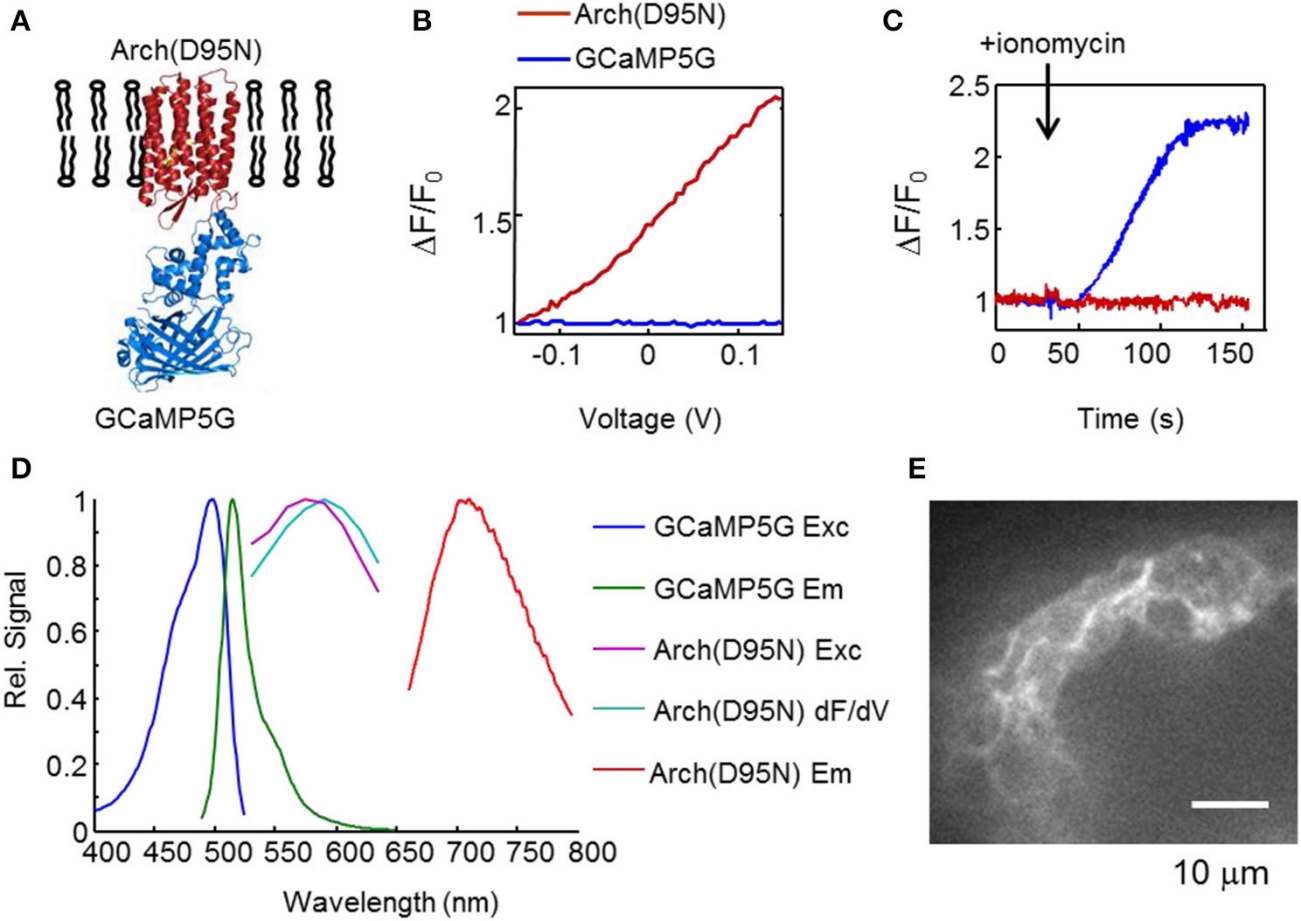
**CaViar reporter for simultaneous measurement of voltage and Ca^2+^**. **(A)** Structure of CaViar: a fusion of the far-red voltage indicator Arch(D95N) with a blue/green calcium indicator (GCaMP5G). **(B)** Fluorescence response of CaViar to variations in membrane voltage in HEK cells. **(C)** Temporal response of CaViar to ionomycin which gradually raised the intracellular calcium concentration from ~100 nM to 1.8 mM. In **(B**,**C)**, the red trace indicates fluorescence of Arch(D95N), and the blue trace indicates fluorescence of GCaMP5G. **(D)** Spectral properties of GCaMP5G (courtesy of Douglas Kim, Janelia Farm) and Arch(D95N). The excitation and emission spectra of Arch(D95N) were measured in an epifluorescence microscope with laser excitation and an imaging spectrometer. The wavelength range of the excitation spectra of Arch(D95N) was limited by available laser lines. The blue (488 nm) light used to excite GCaMP5G induced negligible spurious excitation of Arch(D95N), a consequence of the vastly higher illumination intensity needed to excite Arch(D95N) than to excite GCaMP5G. In Arch(D95N), the voltage-sensitivity spectrum (dF/dV) is slightly red-shifted relative to the fluorescence excitation spectrum. **(E)** Optical section of zebrafish heart expressing CaViar, showing localization of the reporter to cell membranes.

The combined Ca^2+^ and voltage indicator (CaViar) comprised a fusion, Arch(D95N)-GCaMP5G (Figure 2A). To avoid spectral crosstalk, the dual-function indicator made use of the non-overlapping spectra of Arch(D95N) (exc. 594–640 nm, em. 710 nm) and GCaMP5G (exc. 488 nm, em. 510 nm) (Akerboom et al., 2012). We simultaneously monitored the fluorescence of Arch(D95N) and GCaMP5G in HEK cells as a function of steady state membrane voltage (Figure 2B). The fluorescence of Arch(D95N) increased roughly linearly with membrane voltage between −150 and +150 mV, with no detectable crosstalk of voltage into the GCaMP5G channel. We tested the response of the indicators to an increase in [Ca^2+^] (Figure 2C). Intact HEK cells expressing CaViar were permeabilized to Ca^2+^ with ionomycin, gradually raising internal [Ca^2+^] to 1.8 mM. Fluorescence of GCaMP5G increased by 225%, while fluorescence of Arch(D95N) did not undergo a detectable change. The excitation and emission spectra of the two indicators did not overlap (Figure 2D), enabling independent optical measurements of each. We found that the 15 amino acid linker was necessary to avoid interactions between the chromophores in Arch(D95N) and GCaMP5G. In constructs where a fluorescent protein and Arch derivative are fused in closer proximity, non-radiative energy transfer from the fluorescent protein to the Arch can lead to voltage-dependent modulation of the fluorescence of the fluorescent protein (Zou et al., 2014). Figures 2B,C show no such optical crosstalk with the long linkers used here. Thus, each indicator reported its intended modality without crosstalk from the other modality.

### Simultaneous Ca^2+^ and voltage imaging in zebrafish hearts

**Figure 3:**
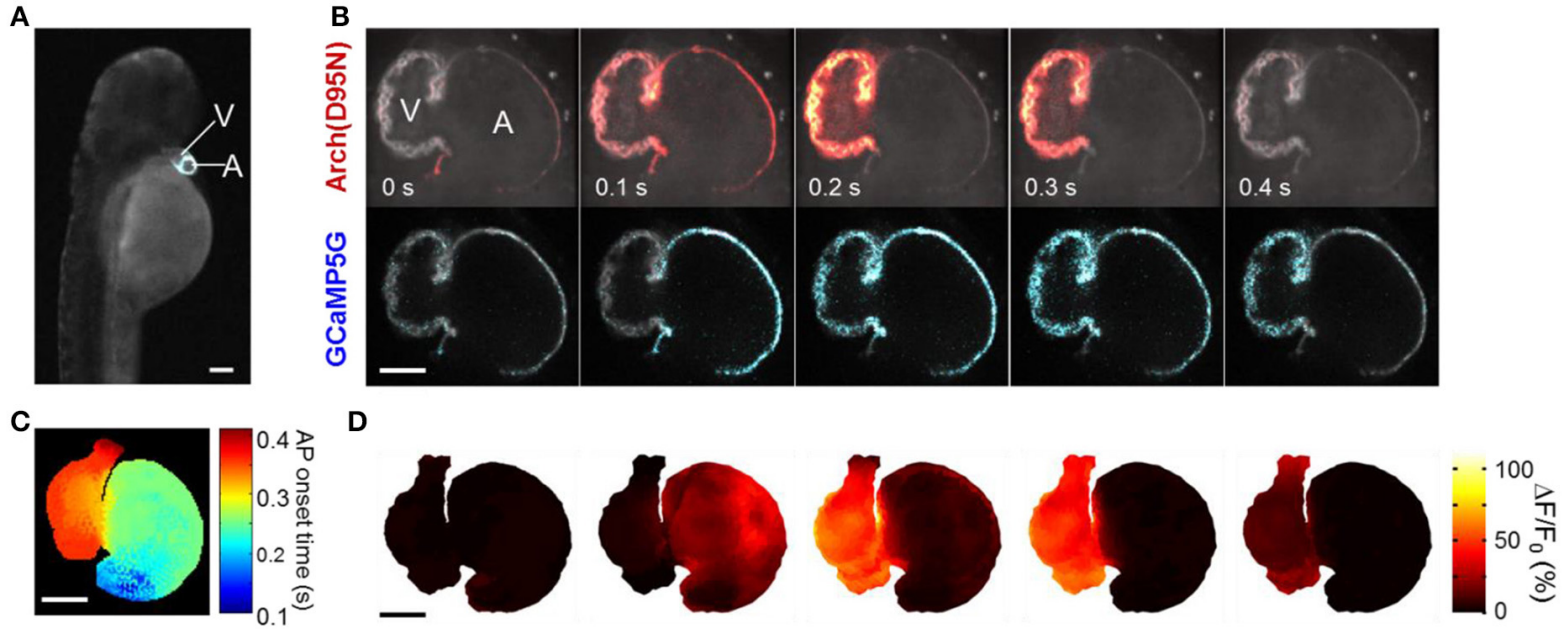
**Mapping simultaneous *V*_m_ and Ca^2+^ in the zebrafish heart *in vivo***. **(A)** Fluorescence of GCaMP5G in a fish expressing CaViar under the *cmlc*2 promoter (Supplementary Movie 1). **(B)** Single optical section of a zebrafish heart expressing CaViar at 4 dpf (Supplementary Movie 2). Cell membranes fluoresced in both the voltage channel (*top*) and the Ca^2+^ channel (*bottom*) as the AP propagated from the atrium to the ventricle. **(C)** Map of AP isochrones overlaid on a three-dimensional reconstruction of the heart. AP onsets were measured as time to reach 5% of maximum on the rising edge, with sub-frame timing achieved through spline interpolation. **(D)** Three-dimensional reconstruction of the electrical AP (Supplementary Movie 3). Scale bars in **(A**–**D)** 50 μm.

We used CaViar to map simultaneous Ca^2+^ and voltage propagation in the embryonic zebrafish heart as a function of developmental stage. High magnification confocal images of zebrafish hearts expressing the indicators showed good localization to the plasma membrane, and very little intracellular fluorescence (Figure 2E). We generated a transgenic zebrafish line expressing CaViar under control of the *cmlc*2 promoter (Figure 3A, Supplementary Movie 1). Fish developed normally and beginning at 24 h post fertilization (hpf) fish showed cardiac-localized fluorescence in both the GCaMP5G and Arch(D95N) channels. At 36 hpf, contractions were visible in the developing heart tube and voltage and Ca^2+^ waveforms could be recorded from the atrium and ventricle using the modified spinning disk confocal microscope. For fish younger than 48 hpf, supplemental retinaldehyde was added prior to imaging to increase fluorescence of Arch(D95N) (see Materials and Methods). Fish treated with supplemental retinaldehyde could be released from the agar and developed normally. After 48 hpf, endogenously produced retinaldehyde was sufficient to saturate the Arch(D95N) binding sites and no supplemental retinaldehyde was added.

We recorded dual-channel fluorescence movies of optical sections of the heart in a fish at 102 hpf. For quantitative measurements, fish were soaked in blebbistatin (10 μM) prior to imaging to eliminate contraction and associated motion artifacts. Due to passive diffusion of oxygen through the tissues, zebrafish do not require a contracting heart to survive the first few days of embryonic development (Bakkers, 2011). Flashes of fluorescence occurred in synchrony with the heartbeat, with near infrared fluorescence emission indicating membrane voltage (*V*_m_) and green fluorescence emission indicating [Ca^2+^] (Supplementary Movie 2). Action potentials waveforms were clearly resolved, with an average Arch(D95N) ΔF/F of ~70% and GCaMP5 ΔF/F of 35%. Some patches in the ventricle reported an Arch(D95N) ΔF/F as high as 110%. The fluorescence responses of Arch(D95N) and GCaMP5 in the atrium averaged ~45 and 55%, respectively. Values of ΔF/F are heavily influenced by the position and orientation of the fish, and by uncontrolled sources of background autofluorescence. Thus, these values do not translate directly into changes in voltage. Our analysis below focuses on action potential waveforms and drug-induced changes in signal amplitude, both of which are robust to these sources of variation.

With blebbistatin, it was possible to observe the propagation of voltage and Ca^2+^ waves across a single plane of the heart (Figure 3B). We generated a three-dimensional reconstruction of voltage propagation (Figures 3C,D, Supplementary Movie 3) which clearly showed the AP originating in the atrium, spreading slowly across the atrioventricular (AV) canal, and rapidly spanning the ventricle.

Fish were maintained on the microscope for up to 6 h without apparent damage. After imaging sessions, fish were released from the agarose and allowed to recover in E3 medium. Voltage and calcium imaging the following day showed normal mechanical, electrical, and calcium heart function, indicating that treatments with blebbistatin and retinaldehyde were fully reversible, and that mounting and imaging caused no apparent damage to fish health.

### Probing zebrafish cardiac maturation via pharmacology

**Figure 4:**
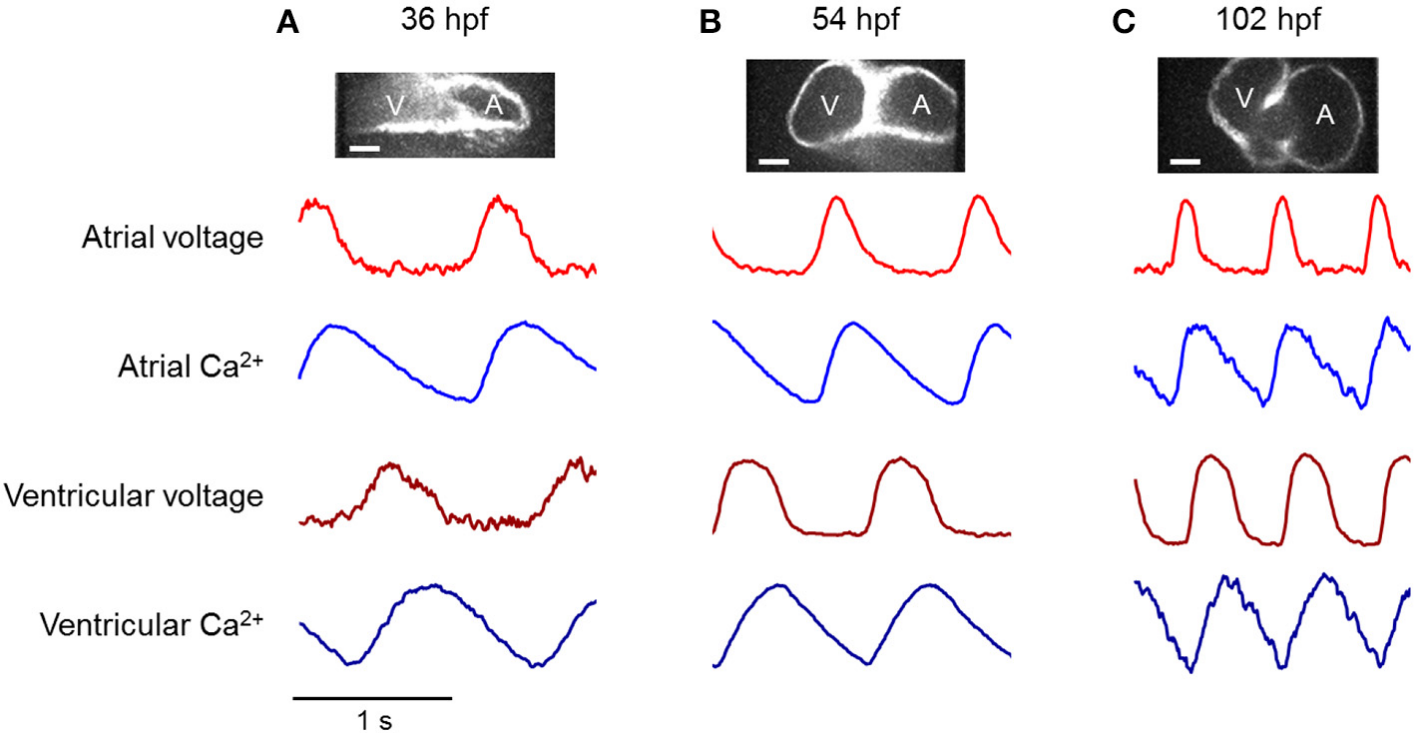
**Voltage and calcium transients of the atrium and ventricle in an embryonic zebrafish heart during development**. **Top**: Fluorescence images of zebrafish hearts expressing CaViar showed transition of the heart tube into two chambers. Scale bars 50 μm. **Bottom**: Dual-wavelength imaging of CaViar reported voltage (*red*), and calcium (*blue*) transients in the atrium (*lighter shade*) and ventricle (*darker shade*) at **(A)** 36 hpf, **(B)** 54 hpf, **(C)** 102 hpf. At 36 hpf, low expression of Arch(D95N) led to higher noise than at other time-points. Blue illumination intensity was minimized at all time-points to minimize photo-inactivation of blebbistatin. This occasionally led to increased noise in the GCaMP5G fluorescence (e.g., at 102 hpf).

We charted the spatial and temporal development of the voltage and Ca^2+^ patterns at 36, 54, and 102 hpf, using new fish at each time point (Figure 4). The heart showed distinct atrial and ventricular AP waveforms as early as 36 hpf (Figure 4A), though the wave propagation was peristaltic with neither a clear electrical nor morphological boundary between the chambers (Tu and Chi, 2012). The electrical APs recorded optically *in vivo* were similar to previous reports of patch clamp measurements on explanted hearts (Chi et al., 2008; Nemtsas et al., 2010). The Ca^2+^ dynamics also varied markedly along the heart tube, with a faster rise and slower decay in the atrium than in the ventricle (Figure 4B), consistent with earlier reports (Chi et al., 2008). By 54 hpf, the delay in electrical propagation at the AV canal was clearly visible, and by 102 hpf the APs in the atrium and ventricle occurred as two clearly resolved beats (Figure 4C).

**Figure 5:**
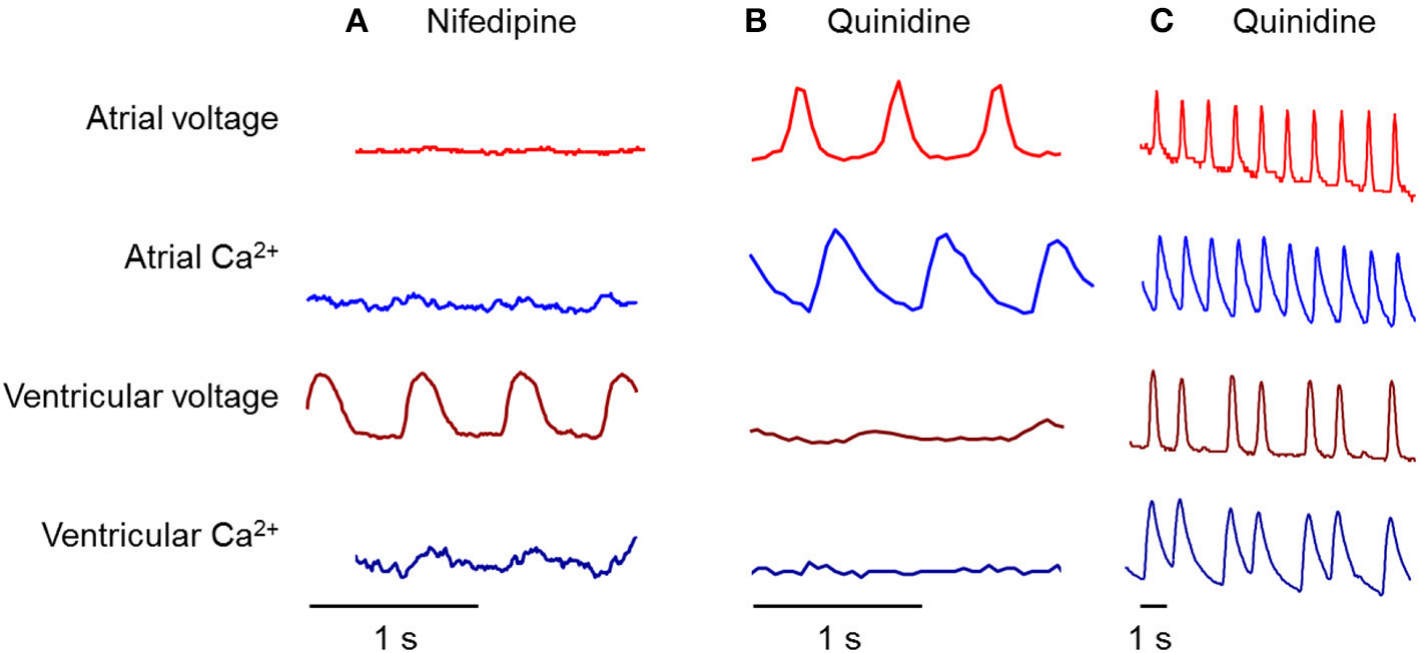
**Differential effects of nifedipine and quinidine on atrial and ventricular chambers at 90–102 hpf**. **(A)** Nifedipine, an L-type calcium channel blocker, largely suppressed calcium transients in both chambers, but only suppressed atrial voltage. **(B)** Quinidine, a blocker of the fast inward sodium current, largely suppressed voltage and calcium transients in the ventricle, but did not affect either transient in the atrium. **(C)** Quinidine sometimes showed partial suppression of ventricular activity, leading to an atrial-ventricular block.

**Figure 6:**
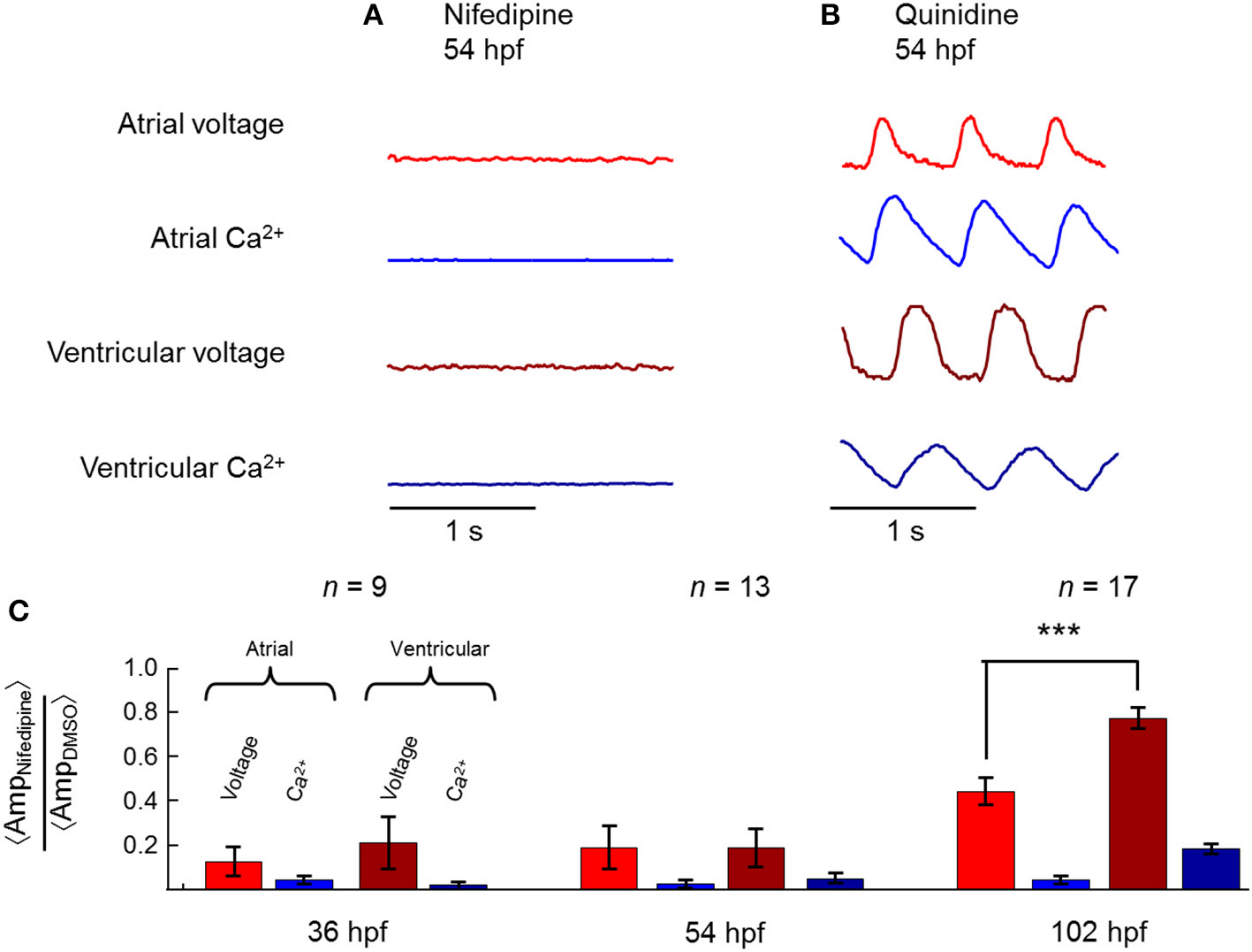
**Differential effects of nifedipine on atrial and ventricular chambers during development**. **(A)** At 54 hpf, nifedipine reversibly suppressed voltage and Ca^2+^ dynamics in both chambers, while **(B)** quinidine had no effect. **(C)** Mean amplitude of the beats in the indicated modality (voltage or Ca^2+^) in the presence of nifedipine, normalized by mean amplitude in the presence of 0.1% DMSO (vehicle control). At 36 and 54 hpf, voltage and Ca^2+^ dynamics were suppressed by nifedipine in both atrium and ventricle. By 102 hpf, calcium activity was still largely suppressed by nifedipine, while voltage dynamics were only partially suppressed, more so in the atrium than in the ventricle. ^***^p = 8.9 × 10^−5^.

**Figure 7:**
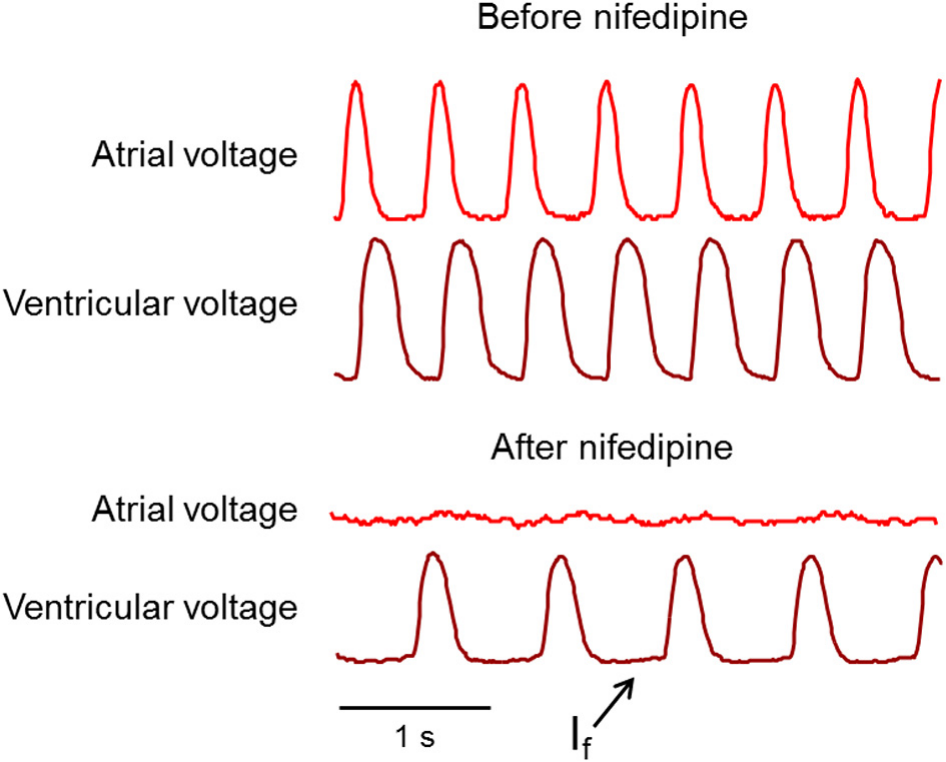
**Appearance of a ventricular funny current, I_f_, after nifedipine treatment**. **Top**: Before nifedipine, ventricular voltage decreased during phase 4 of the AP. **Bottom**: After nifedipine ventricular voltage slightly increased during phase 4, indicating a transition to ventricular pacing.

It was previously shown that in the adult zebrafish heart, the AP is dominated by a Na^+^ current in both chambers and that an L-type calcium channel blocker, nifedipine, does not block the AP (Nemtsas et al., 2010). We asked whether this was true in embryonic zebrafish as well. We examined the effects of nifedipine (10 μM), and a sodium channel blocker, quinidine (10–200 μM), on voltage and Ca^2+^ dynamics. New fish were used at each time point. Figure 5 contains representative traces showing the effect of the drugs on the voltage and Ca^2+^ dynamics, in the atrium and the ventricle, at 90–102 hpf. In all figures showing pharmacological perturbations (Figures 5–7), the amplitude of each voltage and calcium trace is scaled relative to its value in the heart prior to addition of the drug. Thus, the vertical scale represents fractional change in amplitude relative to the untreated heart.

Remarkably, the ventricle and atrium showed strongly divergent responses to nifedipine (Figure 5A). As anticipated for a Ca^2+^ channel blocker, nifedipine largely eliminated the Ca^2+^ signal in both chambers. The residual Ca^2+^ transient in the ventricle may be due to incomplete block of the ventricular L-type Ca^2+^ channels, or due to an additional Ca^2+^-transport pathway. The NCX Na^+^/Ca^2+^ exchanger is electrogenic (it imports three Na^+^ for every Ca^2+^ that it exports) and thus Ca^2+^ export can be slowed or even reversed at depolarizing voltages (Cohen and Venkatachalam, 2014). Presently available data do not distinguish these possible explanations for residual Ca^2+^ dynamics. Surprisingly, nifedipine eliminated the electrical AP in the atrium, but not the ventricle. In contrast, in some instances quinidine blocked the electrical AP in the ventricle, but not the atrium (Figure 5B). When quinidine blocked the electrical AP, it also blocked the Ca^2+^ signal. In other instances quinidine induced a 1:3 AV block (Figure 5C).

At earlier times in development (54 hpf), nifedipine largely suppressed the electrical APs in both chambers (Figure 6A). This suppression was not due to fish death, because upon wash-out of nifedipine the heart re-started. At 54 hpf, quinidine had no observable effect (Figure 6B).

Figure 6C shows summary statistics for the effects of nifedipine at three times in development. At all developmental stages, nifedipine largely suppressed Ca^2+^ transients in both chambers. At 36 and 54 hpf, nifedipine largely suppressed the electrical AP in both chambers as well. However, at 102 hpf, the mean ventricular AP retained 75% of its initial amplitude after addition of nifedipine (range 50–112%; *n* = 17 fish). In contrast, nifedipine eliminated the atrial AP in 4 of 17 fish (<20% of initial amplitude), and partially suppressed the atrial AP (35–80% of initial amplitude) in the remainder fish. The differential effect of nifedipine on AP amplitude in the two chambers was pronounced (*p* = 8.9 × 10^−5^).

Prior to drug addition, none of the ventricular waveforms showed a depolarizing “funny current” during diastole, consistent with a non-ventricular pacemaker. In fish where the atrial AP was suppressed entirely by nifedipine, the ventricular cells showed a significantly slowed beat rate (mean 104 bpm before drug vs. mean 70 bpm after drug, *n* = 4 fish) and a diastolic depolarization (Figure 7). Thus, by 4 dpf, the zebrafish ventricle is capable of autonomous pacing, while at earlier times it is not.

## Discussion

A variety of techniques have been developed for three-dimensional imaging in intact tissue. Two-photon fluorescence imaging has been particularly effective in Ca^2+^ imaging in brain slice and in tissue (Chen et al., 2013), although the limited speed of scanning systems prevents imaging of large fields of view with high frame rates. Selective plane illumination microscopies (SPIM) have been highly effective for imaging in zebrafish heart (Huisken et al., 2004; Arrenberg et al., 2010; Weber and Huisken, 2011) and brain (Ahrens et al., 2013). A key merit of the light-sheet technique is that it minimizes optical exposure of sample planes above and below the focus, thereby minimizing photobleaching and phototoxicity. Spinning disk confocal and light sheet techniques have similar time resolution, ultimately limited either by shot noise or by the frame rate of the camera. While the data presented in this report was acquired at exposure times of 8 ms or longer, we found that the imaging system could run at down to 2 ms/frame with adequate signal levels. The spinning disk technique has the merit of only requiring optical access to the sample from one direction. While access from two directions is not a constraint for zebrafish, unidirectional optical access is convenient for imaging brain slices or larger tissue samples. Future work will focus on development of improved software tools for quantifying fluorescence dynamics in a moving tissue so that blebbistatin is not needed.

Voltage imaging with Arch(D95N) remains technically challenging, principally due to the very dim fluorescence of this construct. Recently, several dramatically improved GEVIs have shown promise for voltage imaging *in vivo*. The zebrafish heart is a natural initial target due to its optical accessibility and comparatively slow dynamics relative to neural firing. The GFP-based ASAP1 reporter (St-Pierre et al., 2014) reports membrane voltage with a sensitivity of ΔF/F = −30% per 100 mV and a response time of 2–3 ms at room temperature. ArcLight (Jin et al., 2012), also based on GFP, has similar sensitivity, but has a complex multi-exponential response with a half-response time of ~100 ms at room temperature. Both ASAP1 and ArcLight show non-linear relations of fluorescence to voltage, with greatest sensitivity near −70 mV.

A recently developed mutant of Arch, termed QuasAr2, has a sensitivity of 90% ΔF/F per 100 mV and a 1 ms response time at room temperature. A related GEVI, QuasAr1, has a sensitivity of 32% per 100 mV and a response time of <50 μs at room temperature (Hochbaum et al., 2014). These new rhodopsin-based GEVIs are much brighter than the first generation of Arch-based reporters, including Arch(D95N), but remain 30–80-fold dimmer than the GFP-based GEVIs. However, the Arch-based GEVIs show greatly enhanced photostability, and at the red wavelengths used for imaging Arch and its variants, there is significantly less background autofluorescence and less phototoxicity than at the blue wavelengths used for imaging GFP. In a side-by-side comparison of QuasAr2 with ArcLight, QuasAr2 reported neuronal action potentials with higher signal-to-noise ratio, *in vitro* and *in vivo* (Hochbaum et al., 2014).

Recent efforts have sought to improve the brightness of rhodopsin-based GEVIs by appending a fluorescent protein fusion, whose fluorescence is selectively quenched by the rhodopsin in a voltage-dependent manner, a phenomenon termed electrochromic FRET (eFRET) (Gong et al., 2014; Zou et al., 2014). To-date the eFRET-based GEVIs have not reached the level of speed, sensitivity, or signal-to-noise ratio found in direct QuasAr fluorescence. All of the GEVIs described above have been tested in rodent brain slice and have shown promise for *in vivo* voltage imaging. Only the rhodopsin-based GEVIs have a sufficiently far-red excitation spectrum to be paired in a crosstalk-free manner with GFP-based reporters of Ca^2+^ and other modalities.

Ca^2+^ indicators with improved sensitivity and kinetics (GCaMP6f) have also recently been reported (Chen et al., 2013). These improved indicators open the possibility to make transgenic fish, mouse, and iPSC lines stably expressing multi-function reporters. The Ca^2+^ reporter used here, could readily be replaced by any other GFP-based genetically encoded reporter. Such tools exist to probe pH, calcium, ATP, NADH, cAMP, glutamate, reactive oxygen species, several redox potentials, activity of kinases and phosphatases, and many other modalities (Hung et al., 2011; Mehta and Zhang, 2011; Depry et al., 2013; Tantama et al., 2013).

Through simultaneous voltage- and Ca^2+^ imaging and pharmacological perturbations, we established that an inward Ca^2+^ flux is required to trigger electrical action potentials in the immature zebrafish heart (<3 dpf). Blockage of the L-type Ca^2+^ channel with nifedipine suppressed voltage and Ca^2+^ dynamics in both chambers. This result contrasts starkly with the adult zebrafish (and human) heart, where a Na^+^ current regulates the upstroke of the action potential. In the adult heart, nifedipine does not suppress electrical activity.

**Figure 8:**
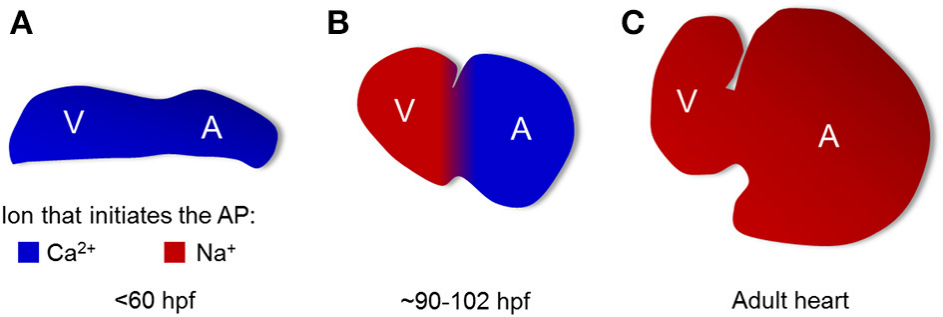
**Maturation of cardiac action potential occurs in a chamber-specific manner**. Model of ionic currents responsible for AP upstroke at different times in development. **(A)** At times <60 hpf, Ca^2+^ initiates the action potential in both chambers. **(B)** At an intermediate times (90–102 hpf), the atrial AP is driven by Ca^2+^ while the ventricular AP is driven by Na^+^. **(C)** In the adult, the AP upstroke in both chambers is driven by Na^+^.

Thus, there must be a transitional phase from immature Ca^2+^-regulated activity (Figure 8A) to mature Na^+^-regulated activity (Figure 8C). We showed that this transition occurs differentially in the two chambers: in the ventricle around 3 dpf and in the atrium around 4 dpf (Figure 8B). Partial suppression of the AP by quinidine in the ventricle, but not the atrium, at 90–102 hpf confirms the transition to a Na^+^-dominated AP upstroke.

Detection of this transition required simultaneous observation of the voltage and Ca^2+^ dynamics in the intact heart. Our results further highlight the importance of working with cardiomyocytes of well-defined subtype and developmental stage for cardiac drug testing—these parameters can have significant effects on drug responses.

The ability to measure voltage and Ca^2+^ simultaneously *in vivo* in the developing zebrafish heart opens the possibility to study the effects of genetic and pharmacological perturbations on development, on excitation-contraction coupling, and on Ca^2+^ handling. Studies on voltage and Ca^2+^ imaging during zebrafish cardiac regeneration would reveal the nature of electrical coupling to cells undergoing transdifferentiation (Zhang et al., 2013). Comparisons of voltage and Ca^2+^ dynamics with and without contraction (modulated via blebbistatin or *silent heart* mutation) enables studies on mechanisms of mechano-electrical feedback *in vivo* (Werdich et al., 2012).

## Sources of funding

This work was supported by the Harvard Center for Brain Science, ONR grant N000141110-549, NIH grants 1-R01-EB012498-01 and New Innovator grant 1-DP2-OD007428, the Harvard/MIT Joint Research Grants Program in Basic Neuroscience, a Helen Hay Whitney Postdoctoral Fellowship (AD), and Charles A. King Trust Postdoctoral Fellowship (AD).

### Conflict of interest statement

AEC and JMK are co-founders of Q-State Biosciences.

## References

[B1] Ahrens M. B. Orger M. B. Robson D. N. Li J. M. Keller P. J. (2013). Whole-brain functional imaging at cellular resolution using light-sheet microscopy. Nat. Methods 10, 413–420 10.1038/nmeth.2434 23524393

[B2] Akerboom J. Chen T. W. Wardill T. J. Tian L. Marvin J. S. Mutlu S. (2012). Optimization of a GCaMP calcium indicator for neural activity imaging. J. Neurosci. 32, 13819–13840 10.1523/JNEUROSCI.2601-12.2012 23035093 PMC3482105

[B3] Arrenberg A. B. Stainier D. Y. R. Baier H. Huisken J. (2010). Optogenetic control of cardiac function. Science 330, 971 10.1126/science.1195929 21071670

[B4] Bakkers J. (2011). Zebrafish as a model to study cardiac development and human cardiac disease. Cardiovasc. Res. 91, 279–288 10.1093/cvr/cvr098 21602174 PMC3125074

[B5] Chen T. W. Wardill T. J. Sun Y. Pulver S. R. Renninger S. L. Baohan A. (2013). Ultrasensitive fluorescent proteins for imaging neuronal activity. Nature 499, 295–300 10.1038/nature12354 23868258 PMC3777791

[B6] Chi N. C. Shaw R. M. Jungblut B. Huisken J. Ferrer T. Arnaout R. (2008). Genetic and physiologic dissection of the vertebrate cardiac conduction system. PLoS Biol. 6:e109 10.1371/journal.pbio.0060109 18479184 PMC2430899

[B7] Chow B. Y. Han X. Dobry A. S. Qian X. Chuong A. S. Li M. (2010). High-performance genetically targetable optical neural silencing by light-driven proton pumps. Nature 463, 98–102 10.1038/nature08652 20054397 PMC2939492

[B8] Cohen A. E. Venkatachalam V. (2014). Bringing bioelectricity to light. Annu. Rev. Biophys. 43, 211–232 10.1146/annurev-biophys-051013-022717 24773017

[B9] Depry C. Mehta S. Zhang J. (2013). Multiplexed visualization of dynamic signaling networks using genetically encoded fluorescent protein-based biosensors. Pflügers Arch. 465, 373–381 10.1007/s00424-012-1175-y 23138230 PMC3584185

[B10] Entcheva E. Bien H. (2006). Macroscopic optical mapping of excitation in cardiac cell networks with ultra-high spatiotemporal resolution. Prog. Biophys. Mol. Biol. 92, 232–257 10.1016/j.pbiomolbio.2005.10.003 16330086

[B11] Fang Q. Boas D. A. (2009). Tetrahedral mesh generation from volumetric binary and grayscale images. IEEE Int. Symp. Biomed. Imaging 1142–1145 10.1109/ISBI.2009.5193259

[B12] Gibson D. G. Young L. Chuang R. Y. Venter J. C. Hutchison C. A. Smith H. O. (2009). Enzymatic assembly of DNA molecules up to several hundred kilobases. Nat. Methods 6, 343–345 10.1038/nmeth.1318 19363495

[B13] Gong Y. Wagner M. J. Li J. Z. Schnitzer M. J. (2014). Imaging neural spiking in brain tissue using FRET-opsin protein voltage sensors. Nat. Commun. 5, e3674 10.1038/ncomms4674 24755708 PMC4247277

[B14] Hochbaum D. R. Zhao Y. Farhi S. Klapoetke N. Werley C. A. Kapoor V. (2014). All-optical electrophysiology in mammalian neurons using engineered microbial rhodopsins. Nat. Methods 11, 825–833 10.1038/nmeth.3000 24952910 PMC4117813

[B15] Huisken J. Swoger J. Del Bene F. Wittbrodt J. Stelzer E. H. (2004). Optical sectioning deep inside live embryos by selective plane illumination microscopy. Science 305, 1007–1009 10.1126/science.1100035 15310904

[B16] Hung Y. P. Albeck J. G. Tantama M. Yellen G. (2011). Imaging cytosolic NADH-NAD+ redox state with a genetically encoded fluorescent biosensor. Cell Metab. 14, 545–554 10.1016/j.cmet.2011.08.012 21982714 PMC3190165

[B17] Jin L. Han Z. Platisa J. Wooltorton J. R. A. Cohen L. B. Pieribone V. A. (2012). Single action potentials and subthreshold electrical events imaged in neurons with a fluorescent protein voltage probe. Neuron 75, 779–785 10.1016/j.neuron.2012.06.040 22958819 PMC3439164

[B18] Kaestner L. Lipp P. (2011). Screening action potentials: the power of light. Front. Pharmacol. 2:42 10.3389/fphar.2011.00042 21847381 PMC3147179

[B19] Kralj J. M. Douglass A. D. Hochbaum D. R. Maclaurin D. Cohen A. E. (2012). Optical recording of action potentials in mammalian neurons using a microbial rhodopsin. Nat. Methods 9, 90–95 10.1038/nmeth.1782 22120467 PMC3248630

[B20] Kralj J. M. Hochbaum D. R. Douglass A. D. Cohen A. E. (2011). Electrical spiking in escherichia coli probed with a fluorescent voltage indicating protein. Science 333, 345–348 10.1126/science.1204763 21764748

[B21] Maclaurin D. Venkatachalam V. Lee H. Cohen A. E. (2013). Mechanism of voltage-sensitive fluorescence in a microbial rhodopsin. Proc. Natl. Acad. Sci. U.S.A. 110, 5939–5944 10.1073/pnas.1215595110 23530193 PMC3625274

[B22] Mandel Y. Weissman A. Schick R. Barad L. Novak A. Meiry G. (2012). Human embryonic and induced pluripotent stem cell–derived cardiomyocytes exhibit beat rate variability and power-law behavior. Circulation 125, 883–893 10.1161/CIRCULATIONAHA.111.045146 22261196 PMC3697086

[B23] Mehta S. Zhang J. (2011). Reporting from the field: genetically encoded fluorescent reporters uncover signaling dynamics in living biological systems. Annu. Rev. Biochem. 80, 375–401 10.1146/annurev-biochem-060409-093259 21495849 PMC4384825

[B24] Milan D. J. Giokas A. C. Serluca F. C. Peterson R. T. MacRae C. A. (2006). Notch1b and neuregulin are required for specification of central cardiac conduction tissue. Development 133, 1125–1132 10.1242/dev.02279 16481353

[B25] Milan D. J. Kim A. M. Winterfield J. R. Jones I. L. Pfeufer A. Sanna S. (2009). Drug-sensitized zebrafish screen identifies multiple genes, including GINS3, as regulators of myocardial repolarization. Circulation 120, 553–559 10.1161/CIRCULATIONAHA.108.821082 19652097 PMC2771327

[B26] Milan D. J. Peterson T. A. Ruskin J. N. Peterson R. T. MacRae C. A. (2003). Drugs that induce repolarization abnormalities cause bradycardia in zebrafish. Circulation 107, 1355 10.1161/01.CIR.0000061912.88753.87 12642353

[B27] Nemtsas P. Wettwer E. Christ T. Weidinger G. Ravens U. (2010). Adult zebrafish heart as a model for human heart? *an electrophysiological study*. J. Mol. Cell. Cardiol. 48, 161–171 10.1016/j.yjmcc.2009.08.034 19747484

[B28] Panáková D. Werdich A. A. MacRae C. A. (2010). Wnt11 patterns a myocardial electrical gradient through regulation of the L-type Ca^2+^ channel. Nature 466, 874–878 10.1038/nature09249 20657579 PMC2921013

[B29] Salama G. Morad M. (1976). Merocyanine 540 as an optical probe of transmembrane electrical activity in the heart. Science 191, 485–487 10.1126/science.191.4226.485 1082169

[B30] Sehnert A. J. Huq A. Weinstein B. M. Walker C. Fishman M. Stainier D. Y. (2002). Cardiac troponin T is essential in sarcomere assembly and cardiac contractility. Nat. Genet. 31, 106–110 10.1038/ng875 11967535

[B31] St-Pierre F. Marshall J. D. Yang Y. Gong Y. Schnitzer M. J. Lin M. Z. (2014). High-fidelity optical reporting of neuronal electrical activity with an ultrafast fluorescent voltage sensor. Nat. Neurosci. 17, 884–889 10.1038/nn.3709 24755780 PMC4494739

[B32] Tantama M. Martínez-François J. R. Mongeon R. Yellen G. (2013). Imaging energy status in live cells with a fluorescent biosensor of the intracellular ATP-to-ADP ratio. Nat. Commun. 4,e2550 10.1038/ncomms3550 24096541 PMC3852917

[B33] Tsutsui H. Higashijima S. Miyawaki A. Okamura Y. (2010). Visualizing voltage dynamics in zebrafish heart. J. Physiol. 588, 2017–2021 10.1113/jphysiol.2010.189126 20421282 PMC2911208

[B34] Tsutsui H. Karasawa S. Okamura Y. Miyawaki A. (2008). Improving membrane voltage measurements using FRET with new fluorescent proteins. Nat. Methods 5, 683–685 10.1038/nmeth.1235 18622396

[B35] Tu S. Chi N. C. (2012). Zebrafish models in cardiac development and congenital heart birth defects. Differentiation 84, 4–16 10.1016/j.diff.2012.05.005 22704690 PMC4099249

[B36] Weber M. Huisken J. (2011). Light sheet microscopy for real-time developmental biology. Curr. Opin. Gen. Dev. 21, 566–572 10.1016/j.gde.2011.09.009 21963791

[B37] Werdich A. A. Brzezinski A. Jeyaraj D. Ficker E. Wan X. McDermott B. M. (2012). The zebrafish as a novel animal model to study the molecular mechanisms of mechano-electrical feedback in the heart. Prog. Biophys. Mol. Biol. 110, 154–165 10.1016/j.pbiomolbio.2012.07.006 22835662 PMC3663588

[B39] Zhang R. Han P. Yang H. Ouyang K. Lee D. Lin Y. F. (2013). *In vivo* cardiac reprogramming contributes to zebrafish heart regeneration. Nature 498, 497 10.1038/nature12322 23783515 PMC4090927

[B40] Zou P. Zhao Y. Douglass A. D. Hochbaum D. R. Brinks D. Werley C. A. (2014). *Bright and fast* multicoloured *voltage reporters via electrochromic FRET*. Nat. Commun. 5:4625 10.1038/ncomms5625 25118186 PMC4134104

